# A Case Study of Upper Limb Robotic-Assisted Therapy Using the Track-Hold Device

**DOI:** 10.3390/s22031009

**Published:** 2022-01-28

**Authors:** Marco Righi, Massimo Magrini, Cristina Dolciotti, Davide Moroni

**Affiliations:** 1 Institute of Information Science and Technologies “A. Faedo” (ISTI), The Italian National Research Council (CNR), Via G. Moruzzi 1, 56124 Pisa, Italy; massimo.magrini@isti.cnr.it (M.M.); davide.moroni@isti.cnr.it (D.M.); 2 Department of Traslational Research and New Technologies in Medicine and Surgery, University of Pisa, Lungarno Pacinotti, 43, 56126 Pisa, Italy; c.dolciotti@gmail.com

**Keywords:** robotic assisted therapy, motor and cognitive rehabilitation, post-stroke recovery, exergames, physiological computing, activity tracking, motricity index

## Abstract

The Track-Hold System (THS) project, developed in a healthcare facility and therefore in a controlled and protected healthcare environment, contributes to the more general and broad context of Robotic-Assisted Therapy (RAT). RAT represents an advanced and innovative rehabilitation method, both motor and cognitive, and uses active, passive, and facilitating robotic devices. RAT devices can be equipped with sensors to detect and track voluntary and involuntary movements. They can work in synergy with multimedia protocols developed ad hoc to achieve the highest possible level of functional re-education. The THS is based on a passive robotic arm capable of recording and facilitating the movements of the upper limbs. An operational interface completes the device for its use in the clinical setting. In the form of a case study, the researchers conducted the experimentation in the former Tabarracci hospital (Viareggio, Italy). The case study develops a motor and cognitive rehabilitation protocol. The chosen subjects suffered from post-stroke outcomes affecting the right upper limb, including strength deficits, tremors, incoordination, and motor apraxia. During the first stage of the enrolment, the researchers worked with seven patients. The researchers completed the pilot with four patients because three of them got a stroke recurrence. The collaboration with four patients permitted the generation of an enlarged case report to collect preliminary data. The preliminary clinical results of the Track-Hold System Project demonstrated good compliance by patients with robotic-assisted rehabilitation; in particular, patients underwent a gradual path of functional recovery of the upper limb using the implemented interface.

## 1. Introduction

The Track-Hold System (THS) project, developed in a healthcare facility and therefore in a controlled and protected healthcare environment, is carried out in the broader framework of Robotic-Assisted Therapy (RAT). RAT represents an advanced and innovative rehabilitation method, both motor and cognitive, and makes use of active, passive, and facilitating robotic devices. Sensors often equip robotic devices for detecting and tracking both voluntary and involuntary movements, and specially developed multimedia protocols to achieve the highest possible level of functional re-education.

Like conventional rehabilitation methods (e.g., Perfetti Method, Mirror Therapy, Biofeedback, etc.), the RAT requires close collaboration between the members of the Multidisciplinary Team, which in the RAT, besides the Doctor and the Therapist, requires the Physiologist and Biomedical and Computer Engineer [1].

### Track-Hold: A System for Robotic-Assisted Therapy

Track-Hold is a passive robotic arm capable of recording and facilitating the movements of the upper limbs. The robotic device was created in its mechanical and sensor components by the “Wearable Robotics” Laboratory, a spin-off of the Institute of Biorobotics of the Sant’Anna School of Advanced Studies (Pisa), and completed by an operational interface for its use in the clinical field, by the Institute of Science and Technology (ISTI) “Faedo” of the National Research Council (CNR) of Pisa, based on specialist clinical advice.

Thanks to a solution based on levers and weights, the arm allows to move a person’s upper limb with a push from the bottom up, varying the weight. Track-Hold records the movements of the limb of the subject under various conditions and multiple settings. Namely, Track-Hold settings permit the movement of the subject’s limb in the following conditions:

- in the absence of gravitational force or with opposing gravitational pull (thus, the user to keep the arm in balance must apply a downward force);
- in the presence of the natural force of gravitation;
- in an intermediate condition.

The levers, the weights, and the limb constitute a set of scales that allow it to function even in equilibrium conditions. These characteristics, especially the functioning with the limb in weightlessness, make it suitable for the rehabilitation and passive training of subjects with motor difficulties of the upper limbs, resulting from previous ischaemic events, progressive neurological pathologies, and extreme loss of strength—reduction in muscle mass or, again, coordination or difficulty in organizing purposeful movements.

The execution of the limb movements of the patient to be re-educated is facilitated by the passive mechanics of the balance wheel, as the upward thrust allows the patient to move the limb with minimal muscular effort. The clinical evaluation made by the physician is used to adapt the amount of support, determining how much weight should be relieved. The device consists of arms connected by joints. Each of the joints is equipped with a patented precision angle sensor [2].

The integrated Track-Hold technology allows recording the arm’s movements through a two-way association between the actions of the upper limb and those of the robotic arm. The recorded kinematic data are objective and can be used, among other things, to estimate improvements and progress of personalized therapeutic programs.

The manipulator of the device, consisting of a handle, is equipped with a pressure sensor that allows detecting when it is pressed with the hand (for example, by detecting the gesture of gripping objects). The sampling rate of the positions is 100 Hz, which is sufficient for correct motion detection. The device is connected to a computer via a USB interface.

Track-Hold has been equipped with an agent software that acts as an operational interface. CNR-ISTI Signals and Images laboratory (SI-Lab) set up the interface in the laboratory in collaboration with the Clinicians (this activity took place between July 2020 and October 2020). The operational interface offers a wide range of exercises that the patient must perform under the control of the operators.

The research work, which took place in the period indicated above, had among the main objectives the creation of special software which uses the potential of the device technology in response to the clinical-rehabilitation needs.

## 2. State of the Art in Robotic Assisted Therapy (RAT)

Robot Assisted Therapy (RAT) with both passive and active devices, is an innovative form of rehabilitation that enables highly repetitive, intensive, adaptive, and quantifiable physical training. It has been increasingly used to restore loss of motor function, mainly in stroke survivors suffering from an upper limb paresis. Multiple studies collated in a growing number of review articles showed the positive effects on motor impairment, less clearly on functional limitations [1]. Recovering upper limb motor functions has important implications for improving the independence of patients after stroke and also in patients with tetraplegia caused by spinal cord injury, traumatic brain injury, and in patients with impaired function of motor neurons, that characterize certain neurological diseases, such as multiple sclerosis, cerebral palsy, Guillain-Barre syndrome, finally in patients affected by essential tremor, and Parkinson’s disease [1,3,4].

In this context, Robotic Assisted Therapy (RAT) is an innovative approach to upper limb rehabilitation. The RAT involves intensive and repetitive exercise, with interactive and individualized practice, which could also imply the execution of oriented tasks or dual-task paradigms, as was shown in our preliminary work [5].

The RAT encompasses technological solutions, such as computerized control systems, and mechanical devices, with both active and passive functioning, that could promote motor learning [6].

Promoting processes of motor learning and cross-education from unaffected to affected upper limb [7] is the most important goal of rehabilitation, because the recovery of motor functions requires neurophysiological mechanisms.

The devices that implement RAT, such as wearable sensors and free-contact systems for gesture tracking, are highly reliable to capture and measure kinematic and dynamic parameters of the upper limb, such as movement quality, speed, direction, strength and range of joints motion [5,8,9].

Results of previous studies [4,10] showed that RAT could improve motor control, regulating muscle activation patterns, as required for the execution of aimed movements during daily activities. It has also been reported that these improvements of motor skills are increased both in the short and long term [4].

A recent metanalysis [11] shows that RAT, compared to the traditional rehabilitation approach, enhances, with prime evidence, arm motion and muscle strength, improving the motor ability of patients in daily activities, and finally, their quality of life.

Notably, it has been reported that RAT, with individualized and customized protocol of exercises, could improve in post-stroke patients also neurophysiological aspects of the upper limb, mainly enhancing shoulder and elbow range of motion [12,13,14,15,16].

The most important paradigm of RAT is not only to maximize the number of task repetitions but also to increase patient attention and effort as well [17].

Indeed, it is known that repetitive and monotonous exercises provide worse retention of motor skills, compared with alternate training [18].

Furthermore, it has been speculated that RAT could decrease recovery of motor functions if it encourages assuaging since the patients could decrease effort and attention due to the use of adaptive algorithms, i.e., during training with active robotic devices [19].

Indeed, clinical evidence shows that, since learning is error-based, a faster improvement of motor skills may be achieved when the rate of error is increased [20].

Cognitive and motor rehabilitation after stroke and other neurological diseases is firmly based on intensive and repetitive training and task-specific learning for promoting neural reorganization and subsequent recovery [21]. Conventional methods for cognitive and motor rehabilitation of upper limbs, such as paper and pencil tasks performing, still strive to accomplish this therapeutic aim [22].

Moreover, studies with stroke survivors have shown a differential pattern of motor outcomes, depending on the cognitive domain impaired [23].

Rehabilitation methods, based on robotic systems, implemented with exergames, provided positive results in patients with post-stroke disabilities of upper limb [24].

Exergames, used as task-oriented motor functions, have been used in RAT to improve certain cognitive functions, promoting the activity of specific domains, especially those related to executive functions and attention, that regulate decision making, movement programming, and time reaction [25].

As described above, state of the art indicates that the combination of RAT with exergames might have a beneficial outcome. In consideration of our previous work [2] in which a specific suite of exergames was designed for use with the Track Hold System, in this paper, we extend the experimental results by proposing a pilot study and analyzing the impact of our methodology in improving upper limb motricity.

## 3. The Case Study

### 3.1. Design and Objectives

Our study was an extended case report with an enrollment of a small sample of patients.

The enrolled patients signed an informed consent drafted according to the guidelines of the local Ethics Committee, furthermore, the procedures were carried out in accordance with the provisions of the Declaration of Helsinki on clinical trials on humans.

SI-Lab staff, the practitioner in neuro-rehabilitation, and the physiotherapists operating in the ICARE Healthcare Facility of the former Tabarracci hospital (Viareggio) collaborated on the THScase study experimentation.

The case study mainly had the objective of developing a motor and cognitive rehabilitation protocol in subjects with post-stroke outcomes affecting the right upper limb, including strength deficits, tremor, incoordination, and motor apraxia.

### 3.2. Experimental Protocol

The protocol consisted of a 4-week training period for subjects with a motor deficit of the right upper limb; enrolled patients performed guided motor exercises with weight relief using the TrackHold device. Patients performed the proposed tasks following a criterion of progressive difficulty (achievement of a greater number of key points). Furthermore, based on the progressive recovery of muscle strength, weight relief was slowly reduced, clinically assessing each patient’s level of muscle strength session by session.

## 4. Patients and Methods

### 4.1. Patients

Four patients, two men, and two women, completed the minimal proposed tasks proposed in this study. The patients were enrolled after neurological assessment and neuropsychological screening. Patients description, clinical data and anthropometric parameters have been reported in the Table 1). Our patients were in post-stroke subacute stage, with impaired motricity of the right upper limb.

### 4.2. Methods

The extended case report was carried out in a period of three months, during which we ran the experimental procedures. There were 3 phases of the experimentation:

- A phase for the evaluation of subjects eligible for the rehabilitation protocol;
- An execution phase in which THS sessions were carried out;
- A follow-up phasefor the assessment of the clinical outcome.

#### 4.2.1. Stages of the Experimentation

During this phase of the trial, the clinicians evaluated the patients to establish patients’ eligibility in the protocol and estimate the level of disability.

The presence of moderate-to-severe cognitive impairment, assessed by the Mini screening test Mental State Evaluation (MMSE), was considered a fundamental criterion for excluding patients from the present experimental protocol.

To be enrolled, subjects had a score equal or bigger to $\frac{24.3}{30}$ on the MMSE. Indeed, MMSE is the most accepted neuropsychological screening test evaluating cognitive assessment, which is investigated in the principal domains, such as memory, attention, visuospatial orientation, and language. This test contains 30 items and a diagnosis of dementia by a total score lower than $\frac{23}{30}$ is suggested [26].

A clinical and pharmacological anamnesis was then carried out, along with the neurological physical examination and the survey of the main anthropometric parameters (weight, height, body mass index (BMI), lengths, and circumferences of the limb upper right). The clinicians selected patients in acute and subacute post-stroke, veterans of the event, vascular affecting the left hemisphere, with a motor disability of the right upper limb.

Table 1 contains the four subjects enrolled (two women and two men) comprising their clinical and anamnestic characteristics.

The screening phase required a baseline assessment (T_0_) using standardized clinical scales (listed below) to establish the level of muscle strength, motor ability, and spasticity of the upper limb.

After four weeks of training at follow-up (T 1), the clinicians performed the same type of clinical evaluation.

The evaluation scales used are the following:

- MRC scale: assesses the level of motor disability. MRC scale is a grading system and provides the following scores: 0, for paralysis; 1, only a trace or flicker of muscle contraction is seen or felt; 2, muscle movement is possible with gravity eliminated; 3, muscle movement is possible against gravity; 4, muscle strength is reduced but the movement against is possible [27]. Maximum total score for shoulder-elbow-wrist = 55/55 (corresponding to no disability).
- Motor index: evaluates movement against gravity and resistance. Motor Index (or Motricity Index) can be used to assess the motor impairment in a patient who has had a stroke. Tests for each arm start with assessing inch grip using a 2.5 cm cube between the thumb and forefinger. Scores are as follows: 19 points are given if the subject is able to grip cube but not hold it against gravity, 22 points are given if able to hold cube against gravity but not against a weak pull, and 26 points are given if able to hold the cube against a weak pull but strength is weaker than normal. In the second part of the test, an elbow flexion from 90° is required, so that the arm touches the shoulder. Scores are as follows: 14 points are given if movement is seen with the elbow out and the arm horizontal. In the third part of the test, shoulder abduction moving the flexed elbow is required from off the chest. In this case, 19 points are given when the shoulder is abducted to more than 90° beyond the horizontal against gravity but not against resistance [28]. Maximum shoulder-elbow-wrist total score = 99/99 (corresponding to normal movement also against resistance and equivalent to the left side)
- Ashworth scale: assesses the severity of the spasticity. Ashworth scale is the most accepted clinical tool to measure the increase of the muscle tone and spasticity; it is a six-point scale (for upper and lower limbs). Scores range is from 0 to 4, where lower scores represent normal muscle tone [29]. Maximum total score = 4/4 (corresponding to affected part rigid in flexion or extension).

#### 4.2.2. Motor and Cognitive Rehabilitation Protocol: The Track-Task

The exercises carried out with the Track-Task aim to make movements valuable and necessary for the execution of daily life activities.

The movements functional to each exercise, carried out by manipulating the device, are composed of sub-movements, which consist in making a cursor on the screen placed in front of the patient reach key-points, characterized by a particular angle and a position. The movement of the manipulator in the frontal plane (XY) finds an immediate correspondence with the position on the screen (easy to identify even by the patient who is performing the exercises). As for the movement in-depth (along the Z-axis), it was chosen to represent it with a variation in the size of the cursor: pushing the manipulator forward (Z coordinate increase) decreases its size, the object moves away. This metaphor is very intuitive and allows, by diversifying the size of the target key points and consequently their position on the Z-axis, to guide the subject to perform the movements in three dimensions. These features have been explained with great attention to patients in order to obtain the best for the best possible performance.

The Track-Task design allows working in a double-screen configuration. The first large screen faces the patient who has to do the rehabilitation or training session. The second screen faces the operator who enters the patient’s identification data and selects the training according to the planned rehabilitation therapy by selecting suitable buttons on the control panel. The configuration with two screens allows the system to not confuse or distract the patient with the exercise preparation operations.

During the rehabilitation sessions the following data relating to the movements made by the subject were acquired:

- Total time: total time elapsed, in seconds.
- Distance overhead: the percentage that indicates the actual distance traveled by the cursor in space, compared to the minimum possible distance between the key points to be reached.
- LF index, HF index: these data are produced by a frequency analysis on the trajectories. The analysis is very important as it allows to detect some indications of tremors associated with neuromotor and/or neurological disorders. Without going into detail here, we can simply say that those at a relatively low frequency (5–8 Hz) are symptoms of neuromotor problems deriving from problematic damage inherent in the post-stroke phase or parkinsonian-type pathologies. Those with a higher frequency (8–15 Hz) can be classified as systemic or physiological tremors, the extent of which may be a function of the subject’s age. The Track-Task application then performs a frequency analysis of the movement trajectories in order to distinguish these two bands.

The Track-Task has a protocol organized in exercises having increasing duration and difficulty. The proposed exercises guide the patient to perform a functional movement due to its characteristics, as it recalls precise gestures corresponding to daily actions.

Each protocol contains a certain number of tasks; each task is composed of sub-movements divided into segments. The subject has to act its limb forward to some oriented key points placed in three-dimensional space, moving the manipulator and following the cursor’s movements on the monitor.

The Tasks included in the protocols are the following:

1. Pushing a door: the act of pushing a door involves reaching two key points.
2. Turning off a switch: the gesture of turning off a switch involves reaching two key points.
3. Opening the door to the left by grasping the handle: the gesture of opening the door by turning the handle involves reaching three key points.
4. Opening the sliding door of a wardrobe: the gesture of opening a door or sliding door involves reaching three key points.
5. Bring a bottle of water to the mouth: the gesture of bringing the bottle towards the mouth to drink implies the achievement of three key points.
6. Mixing the contents of a container clockwise: the gesture of mixing the contents of a container involves reaching 4 key points.
7. Cleaning a surface or glass: the gesture of cleaning a surface with large movements that go from left to right and vice versa implies the achievement of 7 key points.

The system has three different protocols having increasing duration and difficulty. The subject executes various protocols in rehabilitation training, according to the indication of the clinician. The adopted protocols are based on the assessment of the level of motor skills, strength, and muscle tone that the clinician evaluates in correspondence with pre-established follow-up. More in detail, the three configurable protocols include: (i) a short session composed of three tasks, (ii) a medium one containing four tasks, and (iii) a long one having seven tasks. Before executing each protocol, an additional functionality of the system is used to acquire, under the guidance of the operator, the motor parameters of the patients, namely the “Range Of Movement” of the upper limb joints (ROM).

#### 4.2.3. THS Sessions

The four patients enrolled underwent an overall training period of 4 weeks. A training session preceded the actual training to learn how to use the device correctly.

The robotic arm needs weight for proper use. Clinicians measured the patient’s muscle strength to calculate the weight relief to use with the robotic arm at the start of each training session. Clinicians used a dynamometer and the arm curl test to take the measurements. At the end of the same session, the patient indicated the level of perceived fatigue using a Visual-Analogue Scale (VAS). Each rehabilitation session lasted about 30 min.

#### 4.2.4. Rehabilitation Settings

Each patient performed specific tasks. The clinician chose the task difficulty for each session for each patient with the following criteria:

1. the patient’s subjective motor learning ability;
2. muscle strength recovery of the patient.

The clinician assessed the last point (muscle strength recovery) for each patient at the start of each session using dynamic strength tests:

- Dynamic muscle strength: evaluation of the muscle strength of the upper limb using a dynamometric platform (test time = 10 s);
- Arm curl: This test measures the strength and endurance of the lower limb. The clinician loaded the Track-Hold loaded with 1 kg for the female and 2 kg for the male. During the test, the subject has to perform as many arm curls as possible. The test length is 30 s; the patient executes the test on the side of the dominant arm.

At the end of the session, the clinician assessed the level of psycho-physical fatigue using the index of fatigue by presenting an analogical scale to the subject. The subject uses this analogic scale by pointing to the area corresponding to the perceived fatigue. The score is from 0 to 10.

To obtain weight relief, clinicians used three weights installing them on the Track-Hold device: (1) 976 g, (2) 1429 g, (3) 3894 g.

**Figure 1:**
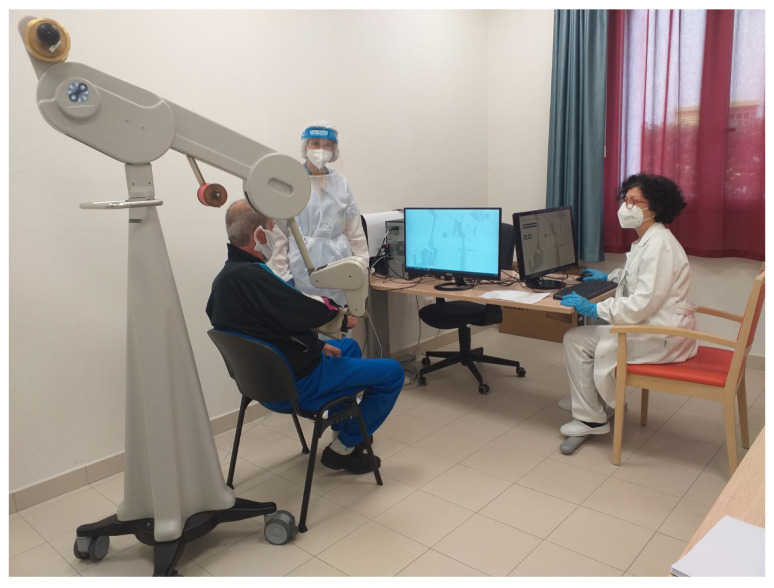
A clinician during the set-up session.

**Figure 2:**
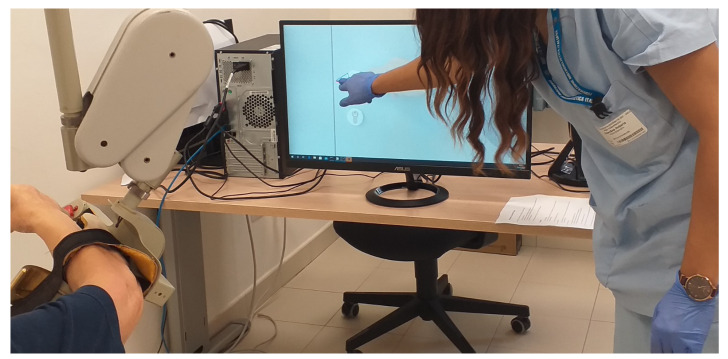
A physiotherapist during the set-up session.

The doctor and the physiotherapist assisted the patient during the rehabilitation setting (Figure 1 and Figure 2).

## 5. Results

### 5.1. Adherence and Compliance of Patients to THS Protocol

#### 5.1.1. Patient 1

Patient 1 participated in 12 rehabilitation sessions, performing a total of 47 tasks. The training led to a visible improvement in dynamic strength from one session to another and consequent progressive reduction in weight relief. Table 2 summarises the recorded results in the Track-Hold sessions of patient 1.

#### 5.1.2. Patient 2

Patient 2 participated in a total of 4 sessions, carrying out 14 tasks. The performance improvement was less noticeable in terms of weight relief, dynamic strength, and perceived fatigue. Table 3 summarises the recorded results in the Track-Hold sessions of patient 2.

#### 5.1.3. Patient 3

Patient 3 participated in 11 rehabilitation sessions, carrying out a total of 35 tasks. The results show a marked reduction in relief from a moderate increase in dynamic strength and a reduction in the VAS index of fatigue score from 6 to 3. Table 4 summarises the recorded results in the Track-Hold sessions of patient 3.

#### 5.1.4. Patient 4

Patient 4 performed seven sessions (an intermediate number) during 28 tasks. The data gained during the tasks evidence an improvement in motor performance and effort tolerance. Table 5 summarises the recorded results in the Track-Hold sessions of patient 4.

### 5.2. Clinical Outcome

The clinical results of the THS experimental protocol show that this type of training, based on the performance of motor tasks on a guided cognitive paradigm and with progressive relief of severity, resulted in a specific functional recovery of the right upper limb in the short term.

This recovery positively affects the acquisition of muscle strength, motor skills, and less perception of physical and mental fatigue in the patient.

Table 6, Table 7 and Table 8 report the scores of the clinical tests performed at T0 (baseline) and repeated at T1 (follow-up) after four weeks of treatment.

THS training seems to have improved the overall motor skill of the right upper limb, particularly elbow and shoulder mobility, as reported in Table 6 (Motricity Index). Table 6 uses the followings evaluation indices:

- scoring method for pliers grip:
  – 0 = no movement
  – 11 = start of pressure, some movements of thumb and forefinger
  – 19 = grip is possible but not against gravity
  – 22 = grip is possible against gravity but not against resistance
  – 26 = grip possible against resistance but weaker than left side
  – 33 = normal socket
- scoring method for elbow bend and shoulder abduction:
  – 0 = no movement
  – 9 = muscle contraction is possible without appreciable movement
  – 14 = movement is possible but not for all ranges of joint motion or against gravity
  – 19 = movement is possible throughout the range of joint motion against gravity but not against resistance
  – 25 = movement is possible against resistance but is weaker than the left side
  – 33 = normal movement

## 6. Conclusions and Future Works

Our preliminary results show that THS training could be effective to improve upper limb motricity in the acute and subacute post-stroke stage. Preliminary clinical results of the Track-Hold System demonstrated good patient compliance with this kind of robotic rehabilitation. The interfaces for users and operators made it possible to establish an effective recovery path for patients who were able to gradually resume (even if not completely) the functions of the upper limb. In the form of exergames, the guiding exercises of varying difficulty made it possible to emulate the gestures of daily activities. Following the exergames, the patients learned the lost movement capacity during the workouts; then, they have reproduced the movements in their daily acts, thus approaching the quality of life before the disease.

Further experimentation is necessary for a large sample of patients to confirm the effectiveness of this cognitive and motor rehabilitation approach.

This case study has shown that exercises carried out using a passive robotic device equipped with weight relief allow partial recovery of the functional capacity of the upper limb even three months after a stroke episode. The next step of this study involves a comparison between this rehabilitation approach and a clinical method that is currently used for the functional recovery of stroke survivors, namely Action Observation Therapy (AOT) [30]. The AOT is mainly based on a process of movements learning: during the rehabilitation sessions, the patient tries to reproduce gestures performed by the physiotherapist, which are always representative of activities of daily life. The method described in the present work could offer advantages compared to AOT, in terms of the accuracy of monitoring and efficacy of the treatment [30].

## Figures and Tables

**Table 1:** Patients description and clinical evaluation.

Patients	Sex	Age	Comorbidity	Terapy	MMSE	BI	H (m)	W (Kg)	ULL R(cm)	Fac r (cm)	Fc r (cm)
1	F	76	hypertension dyslipidemia	antiplatelet agents antihypertensive statin	26	80	1.50	56.3	48	29	23
2	F	78	atrial fibrillation depression	oral anticoagulant antiarrhythmic antidepressant	24	60	1.60	52	51	27	23
1	M	72	hypertension siabetes	oral anticoagulant antiarrhythmic antidepressant	27	75	1.75	70	60	31	24
2	M	69	atrial fibrillation	Antiplatelet agent antiarhytmic	25	80	1.68	72	58	30	25

Legend: MMSE, Mini Mental State Examination (cut off = 24.3/30); h, hight; BI, Barthell Index (w, weight; ullr, right nupper limb right; am, arm circonference; fc, forearm circonference.

**Table 2:** Patient 1 training.

Record	Protocol	Task (Run n.)	Dynamic Muscle Strength [Pre Task]	Arm Curl 1 Kg (n./30 s) [Pre Task]	Weight Compensation (g)	VAS (0–10) [Post Task]
I $\left(T_{0}\right)$	short	3	2450	8	6300	7
II	short	3	2450	8	6300	6
III	short	3	2450	8	5324	6
IV	short	3	2500	9	5324	6
V	medium	4	2500	9	5324	5
VI	medium	4	2500	9	5324	5
VII	medium	4	2730	10	4871	5
VIII	medium	4	2700	10	4871	4
IX	medium	4	2750	10	4871	4
X	medium	4	2800	11	4871	4
XI	medium	4	2800	11	2858	4
XII $\left(T_{1}\right)$	long	7	3105	12	2858	4
	Total Task	47				

**Table 3:** Patient 2 training.

Record	Protocol	Task (Run n.)	Dynamic Muscle Strength [Pre Task]	Arm Curl 1 Kg (n./30 s) [Pre Task]	Weight Compensation (g)	VAS (0–10) [Post Task]
I $\left(T_{0}\right)$	short	3	1600	6	8764	6
II	short	3	1660	6	7788	5
III	medium	4	1700	7	6821	5
IV $\left(T_{1}\right)$	medium	4	1870	7	6821	5
	Total Task	14				

**Table 4:** Patient 4 training.

Record	Protocol	Task (Run n.)	Dynamic Muscle Strength [Pre Task]	Arm Curl 1 Kg (n./30 s) [Pre Task]	Weight Compensation (g)	VAS (0–10) [Post Task]
I $\left(T_{0}\right)$	short	3	3655	10	3894	6
II	short	3	3700	10	3894	6
III	short	3	3895	10	3523	6
IV	medium	4	3900	11	3523	5
V	medium	4	4240	11	2585	5
VI	medium	4	4500	11	2585	4
VII $\left(T_{1}\right)$	long	7	4690	13	2858	4
	Total Task	28				

**Table 5:** Patient 3 training.

Record	Protocol	Task (Run n.)	Dynamic Muscle Strength [Pre Task]	Arm Curl 1 Kg (n./30 s) [Pre Task]	Weight Compensation (g)	VAS (0–10) [Post Task]
I $\left(T_{0}\right)$	short	3	3690	10	5268	6
II	short	3	3690	10	5268	6
III	short	3	3690	10	5268	6
IV	short	3	4000	13	4287	5
V	short	3	4000	13	4287	5
VI	short	3	4000	13	4287	5
VII	medium	4	4025	13	4287	4
VIII	medium	4	4320	13	3904	4
IX	medium	4	4375	13	3904	3
X	medium	4	4870	13	3834	3
XI $\left(T_{1}\right)$	long	7	4950	13	2858	3
	Total Task	35				

**Table 6:** Motricity Index item (MI) for right upper limb.

Item MI	Patient	T_0_	T_1_	Item MI	Patient	T_0_	T_1_
Pliers grip	1	19	26	Shoulder abdution	1	14	19
	2	14	19		2	14	14
	3	22	26		3	14	25
	4	22	26		4	19	25
Elbow flexion	1	14	25	Total score	1	47	70
	2	14	19		2	42	52
	3	14	25		3	50	76
	4	19	25		4	50	75

**Table 7:** Screening and follow-up results.

Patient	DMS T_0_(g)	DMST_1_ (g)	AC T_0_ (n/30 s)	ACT_1_ (n/30 s)	WC T_0_ (g)	WC T_1_ (g)	VAS T_0_ (Score)	VAS T_1_ (Score)	MRC T_0_ (Score)	MRCT_1_ (Score)	MI T_0_ (Score)	MI T_1_ (Score)	AS T_0_ (Score)	AS T_1_ (Score)
1	2450	3105	8	12	6300	2858	7	4	31	47	47	70	1+	1
2	1600	1870	6	7	8764	6821	5	5	24	32	42	52	1	1
3	3690	4950	10	13	5268	2858	6	3	40	48	50	76	1	1
4	3655	4690	10	13	3894	2858	6	4	32	44	50	76	2	1

Legend: DNS, Dynamic Muscle Strenght; AC, Arm Curl; WC, Weight Compensation; VAS, Visuo-Analogue Scale of fatigue; MI, Motricity Index; Ashworth Scale.

**Table 8:** MRC scale item (MRCs) for right upper limb.

Patient	Body Area	Item MRC	$T_{0}$	$T_{1}$	Patient	Body Area	Item MRC	$T_{0}$	$T_{1}$
1	shoulder	shrug	2	4	3	shoulder	shrug	3	4
		abduction	2	4			abduction	3	4
		adduction	2	4			adduction	4	4
		extension	3	3			extension	3	4
		flexion	3	4			flexion	4	4
	elbow	extension	3	4		elbow	extension	2	4
		flexion	3	5			flexion	3	4
	wrist	dorsal extension	3	5		wrist	dorsal extension	4	5
		palmar flexion	4	5			palmar flexion	4	5
		abduction	3	5			abduction	4	5
		adduction	3	5			adduction	4	5
		Total score	31	47			Total score	40	48
2	shoulder	shrug	2	3	4	shoulder	shrug	3	4
		abduction	2	3			abduction	2	4
		adduction	2	3			adduction	3	4
		extension	3	3			extension	3	4
		flexion	2	3			flexion	3	4
	elbow	extension	2	3		elbow	extension	2	4
		flexion	2	2			flexion	3	4
	wrist	dorsal extension	3	3		wrist	dorsal extension	4	4
		palmar flexion	2	3			palmar flexion	3	4
		abduction	2	3			abduction	3	4
		adduction	2	3			adduction	3	4
		Total score	24	32			Total score	32	4
